# Reducing stray radiation with a novel detachable lead arm support in percutaneous coronary intervention

**DOI:** 10.1002/acm2.13763

**Published:** 2022-08-24

**Authors:** Atsushi Fukuda, Nao Ichikawa, Takuma Hayashi, Pei‐Jan P. Lin, Kosuke Matsubara

**Affiliations:** ^1^ Department of Radiological Sciences School of Health Sciences Fukushima Medical University Fukushima Fukushima Japan; ^2^ Department of Radiological Technology Faculty of Health Science Kobe Tokiwa University Kobe Hyogo Japan; ^3^ Department of Radiation Oncology Shiga General Hospital Moriyama Shiga Japan; ^4^ Department of Radiology Virginia Commonwealth University Medical Center Richmond Virginia USA; ^5^ Department of Quantum Medical Technology, Faculty of Health Sciences, Institute of Medical, Pharmaceutical and Health Sciences Kanazawa University Kanazawa Ishikawa Japan

**Keywords:** cardiovascular angiography system, detachable lead arm support, radiation protection, stray radiation

## Abstract

**Background:**

Placing radioprotective devices near patients reduces stray radiation during percutaneous coronary intervention (PCI), a promising technique for treating coronary artery disease. Therefore, lead arm support may effectively reduce occupational radiation dose to cardiologists.

**Purpose:**

We aimed to estimate the reduction of stray radiation using a novel detachable lead arm support (DLAS) in PCI.

**Materials and methods:**

A dedicated cardiovascular angiography system was equipped with the conventional 0.5‐mm lead curtain suspended from the table side rail. The DLAS was developed using an L‐shaped acrylic board and detachable water‐resistant covers encasing the 0.5‐, 0.75‐, or 1.0‐mm lead. The DLAS was placed adjacent to a female anthropomorphic phantom lying on the examination tabletop at the patient entrance reference point. An ionization chamber survey meter was placed 100 cm away from the isocenter to emulate the cardiologist's position. Dose reduction using the L‐shaped acrylic board, DLAS, lead curtain, and their combination each was measured at five heights (80–160 cm in 20‐cm increments) when acquiring cardiac images of the patient phantom with 10 gantry angulations, typical for PCI.

**Results:**

Median dose reductions of stray radiation using the L‐shaped acrylic board were 9.0%, 8.8%, 12.4%, 12.3%, and 6.4% at 80‐, 100‐, 120‐, 140‐, and 160‐cm heights, respectively. Dose reduction using DLAS with a 0.5‐mm lead was almost identical to that using DLAS with 0.75‐ and 1.0‐mm leads; mean dose reductions using these three DLASs increased to 16.2%, 45.1%, 66.0%, 64.2%, and 43.0%, respectively. Similarly, dose reductions using the conventional lead curtain were 95.9%, 95.5%, 83.7%, 26.0%, and 19.6%, respectively. The combination of DLAS with 0.5‐mm lead and lead curtain could increase dose reductions to 96.0%, 95.8%, 93.8%, 71.1%, and 47.1%, respectively.

**Conclusions:**

DLAS reduces stray radiation at 120‐, 140‐, and 160‐cm heights, where the conventional lead curtain provides insufficient protection.

## 1 INTRODUCTION

Percutaneous coronary intervention (PCI) first emerged as a minimally invasive procedure in 1977 and is now recognized as a promising technique for the treatment of coronary artery disease.^1^ Owing to recently developed novel methods and devices, PCI is now being used to treat complex lesions in critically ill patients more than ever before.^2^, ^3^ These new methods are associated with the prolonged and extensive use of fluoroscopy in PCI procedures, which increases occupational radiation dose to cardiologists.^4^ In addition, chronic radiation exposure can lead to increased DNA damage among cardiologists, depending on the length of their professional experience.^5^ Therefore, it is critical to maintain the occupational radiation dose as low as reasonably achievable (the ALARA principle).^6^

Several PCI‐related techniques that reduce occupational radiation doses to cardiologists have been reported in the literature.^7^ The most common strategies are to use a lead curtain suspended from the table side rail and a ceiling‐suspended lead shield. Because these protectors cannot sufficiently reduce stray radiation (scattered X‐rays from interactions with the patient and/or examination table) to negligible levels, cardiologists should wear radioprotective gear, such as protective apron, thyroid collar, and lead glasses, during PCI.^8^

It is useful to place radioprotective devices right next to the patient to reduce stray radiation as effectively as possible. Therefore, a radioprotective drape and lead arm support may be promising tools for reducing the occupational radiation dose to the cardiologist.^9^, ^10^ Therefore, we developed a novel detachable lead arm support (DLAS) using an L‐shaped acrylic board and a detachable water‐resistant cover encasing 0.5‐, 0.75‐, and 1.0‐mm leads. Because the risk of bleeding increases following PCI, the detachable water‐resistant cover enables the disinfection of DLAS with alcohol to avoid any blood contamination before treating a new patient. We further hypothesized that when used in combination with the conventional lead curtain, the DLAS would effectively reduce stray radiation. This study aimed to quantify the dose reduction of stray radiation during PCI using a combination of protectors: the L‐shaped acrylic board, DLAS, and lead curtain.

## 2 METHODS

### 2.1 Cardiovascular angiography system and detachable conventional lead curtain

We used a dedicated cardiovascular angiography system (Infinix Celeve‐i, Canon Medical Systems, Nasu, Japan) equipped with a 200 × 200‐mm^2^ flat‐panel image receptor (FPIR). The system is equipped with a kerma‐area product meter (DIAMENTOR K2S, PTW, Freiburg, Germany), which displays incident air kerma value at the patient entrance reference point (PERP) and the kerma‐area product value according to the IEC 60601‐2‐43.^11^ The PERP is defined at a distance of 15 cm from the isocenter toward the X‐ray tube along the central beam axis.^11^

The system has a detachable conventional 0.5‐mm lead curtain (UT6901, MAVIG GmbH, Munich, Germany) suspended from the table side rail and a ceiling‐suspended 0.5‐mm lead shield (OT25B05, MAVIG GmbH, Munich, Germany).

### 2.2 L‐shaped acrylic board and DLAS

**FIGURE 1:**
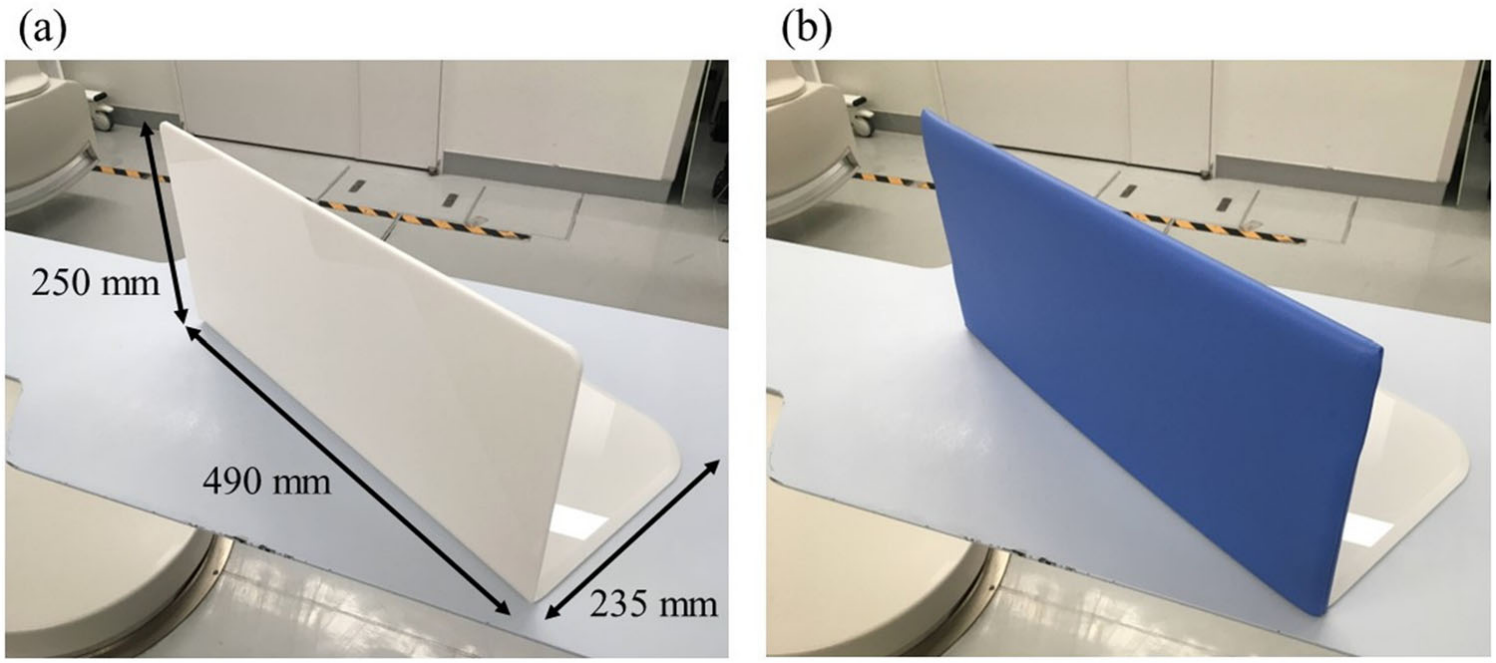
An overview of the detachable lead arm support (DLAS). The left picture (a) shows the L‐shaped acrylic board. The right picture (b) shows the DLAS that consists of the L‐shaped acrylic board and a detachable water‐resistant cover encasing the 0.5‐, 0.75‐, or 1.0‐mm lead.

The DLAS consisted of the L‐shaped acrylic board (250 × 235 × 490 mm, 5‐mm thick) and a detachable water‐resistant cover that encased the 0.5‐, 0.75‐, or 1.0‐mm lead, as shown in Figure 1. As the patient lies on the 5‐cm thick, soft pad on the examination table, the bottom of the DLAS is inserted between the pad and the examination table. Moreover, PCI requires multiple digital acquisitions at different angles for efficient imaging of coronary artery diseases. Based on the clinical circumstances, the size of the DLAS was designed to avoid collision with FPIR.

### 2.3 Ionization chamber survey meter

A 400‐cm^3^ ionization chamber survey meter (LUCREST ICS‐1323, Hitachi Ltd., Tokyo, Japan) was used for stray radiation measurements. It could display the accumulated ambient dose equivalent H*(10) and the respective dose rates ${\dot{H}}^{*}$(10).^12^ The H*(10) represents the operational quantity required for assessing the effective dose for area monitoring. The H*(10) at a point in a radiation field is the dose equivalent that would be produced by the corresponding expanded and aligned field in the International Commission on Radiation Units and Measurement (ICRU) sphere at a depth of 10 mm on the radius opposing the direction of the aligned field.^12^ The H*(10) energy response of the ionization chamber survey meter is within 15% of the true H*(10) over 30 keV to 1.5 MeV.^13^ The relative indication error of the repeated measurements was also reported within 10%.^13^

### 2.4 Measurements of stray radiation and dose reduction

**FIGURE 2:**
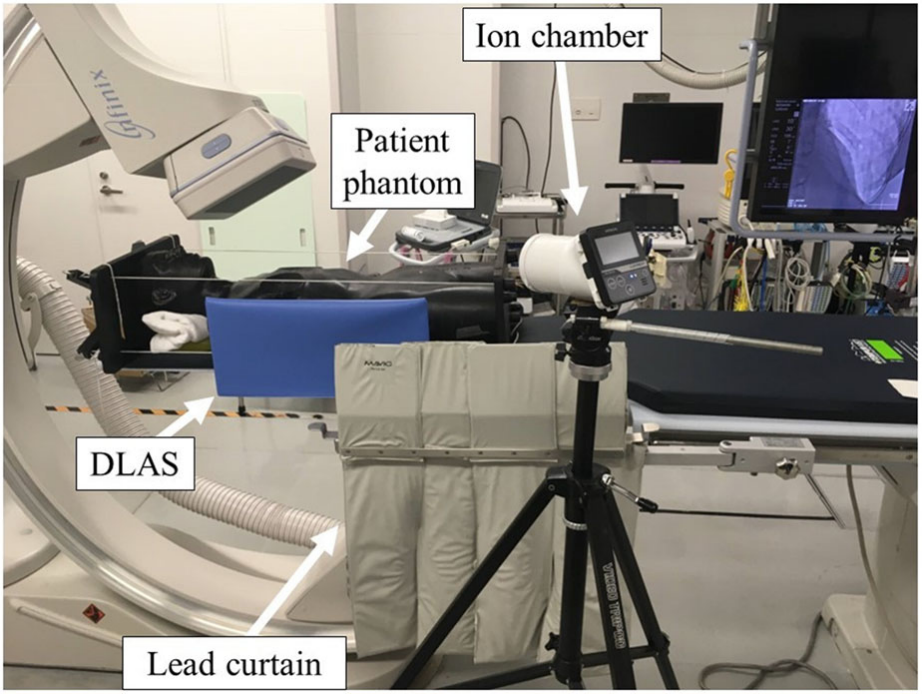
Experimental arrangement for measurements of the H*(10) (photograph). A female anthropomorphic phantom lay on the examination table at the patient entrance reference point (PERP). A 400‐cm^3^ ionization chamber survey meter was placed on the tripod to measure the stray radiation and the dose reduction. DLAS, detachable lead arm support; H*(10), ambient dose equivalent

A female anthropomorphic phantom (Alderson RANDO phantom, Alderson Research Laboratories Inc., Stanford, USA) was placed on the 5‐cm pad on the examination tabletop at the PERP, as shown in Figure 2. After setting the gantry working angle with an FPIR field‐of‐view of 175 × 175 mm^2^, the center of the heart was aligned to the central beam axis in fluoroscopy imaging. Table 1 shows the 10 typical working angles in PCI used in this study.

**TABLE 1:** X‐ray parameters for digital cine acquisitions

	X‐ray parameters						Radiation output
X‐ray tube angulation	SID (cm)	Tube potential (kVp)	Tube current (mA)	Pulse width (ms)	Spectral shaping filter (mmCu)	Focal spot size (mm)	Air kerma rate at PERP (mGy/min)^a^
PA 0°	95	73	144	2.9	0.2	0.8	14.8 ± 1.0
RAO 10°/CAU 30°	105	74	189	3.9	0.2	0.8	27.8 ± 1.3
RAO 30°/CAU 30°	110	74	255	5.1	0.2	0.8	51.7 ± 1.5
RAO 30°	100	74	172	3.5	0.2	0.8	22.5 ± 1.4
RAO 30°/CRA 30°	110	73	227	4.6	0.2	0.8	39.1 ± 2.9
LAO 10°/CRA 30°	105	74	231	4.7	0.2	0.8	41.1 ± 5.1
LAO 30°/CRA 30°	110	74	278	5.5	0.2	0.8	61.2 ± 1.0
LAO 45°/CRA 30°	110	75	433	8.0	0.2	0.8	148 ± 6
LAO 45°	105	73	264	5.2	0.2	0.8	59.4 ± 1.5
LAO 45°/CAU 30°	110	74	413	7.6	0.2	0.8	134 ± 9

Abbreviations: CAU, caudal; CRA, cranial; LAO, left anterior oblique; PA, posterior–anterior; PERP, patient entrance reference point; RAO, right anterior oblique; SID, source–image receptor distance.

^a^ The air kerma rates at PERP (mean ± standard deviation values) were obtained in five measurements as the height of the ionization chamber survey meter was adjusted five times (80‐, 100‐, 120‐, 140‐, and 160‐cm heights).

**FIGURE 3:**
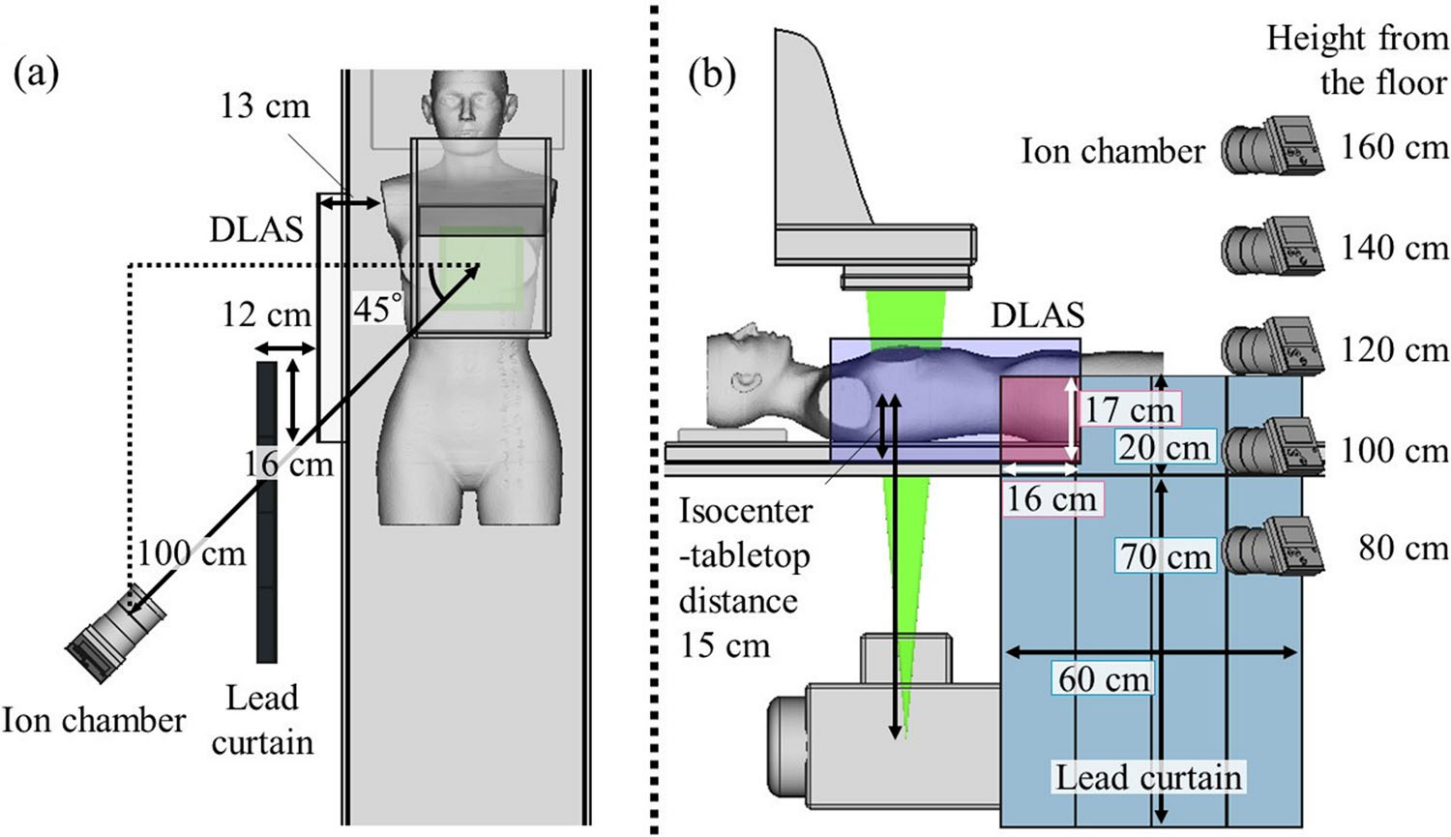
Experimental arrangement for the H*(10) measurements (rendering). (a) The top view of the experimental setup, as prepared using computer‐aided design (CAD) software. An ion chamber survey meter was placed 100 cm away from the isocenter to emulate the cardiologist's position. The distance between detachable lead arm support (DLAS) or the lead curtain and the ionization chamber survey meter remained constant during the measurements because the female anthropomorphic phantom was unnecessary to move (panned) so that the heart centers were shifted in the central beam axis at these working angles. (b) The lateral view of the experimental setup. The height of the ionization chamber survey meter was changed from 80 to 160 cm from the floor in 20‐cm increments. The lead curtain over and under the examination table was 20 and 70 cm, respectively. Furthermore, when using the combination of the DLAS and lead curtain, the lateral and vertical distances of overlap were 16 and 17 cm, respectively (semitransparent red area). H*(10), ambient dose equivalent

A tripod was used to place the ionization chamber survey meter at a distance of 100 cm from the isocenter to emulate the cardiologist's position, as shown in Figure 3. The height of the ionization chamber survey meter varied from 80 to 160 cm in 20‐cm increments.

The L‐shaped acrylic board and DLAS with 0.5‐, 0.75‐, and 1.0‐mm leads were independently placed right next to the female anthropomorphic phantom, with a 13‐cm separation to emulate the right arm space. The detachable conventional 0.5‐mm lead curtain was suspended from the table side rail as required.

As a preliminary study, X‐ray tube angulation was set posterior–anterior (PA), and 30‐s digital cine acquisitions were performed to measure the accumulated H*(10) at a 120‐cm height without any protectors. The digital cine acquisitions were performed using the automatic exposure rate control according to a standard clinical protocol for PCI procedure. The mean (± standard deviation [SD]) and the coefficient of variation were calculated after taking five repeated measurements. The preliminary result showed that the mean ± SD and the coefficient of variation were 3.24 ± 0.07 μSv/30 s and 2.2%, respectively.

Based on the preliminary result, all H*(10) data in this study were obtained using a single measurement because the relative indication error of the ionization chamber survey meter was larger than the coefficient of variation (2.2% < 10%). Source‐to‐image receptor distance (SID), tube potential, tube current, pulse width, spectral shaping filter, focal spot size, and air kerma rate at the PERP were simultaneously recorded. Density equalization filters were not used to prevent any underestimation of the air kerma rate at the PERP.^14^ Furthermore, the accumulated H*(10) using both the DLAS with 0.5‐mm lead and the lead curtain were also measured to determine the combination effect.

Upon the completion of the measurements of the stray radiation with and without protectors, stray radiation dose reductions were calculated as follows:

$$\;R_{w/}=\frac{H^{*}{\left(10\right)}_{w/o}-H^{*}{\left(10\right)}_{w/}}{H^{*}{\left(10\right)}_{w/o}},$$

where *R*_w/_ denotes the dose reductions using the protectors, and H*(10)_w/_ and H*(10)_w/o_ represent H*(10) that was measured with and without the protectors, respectively. The air kerma rate mean ± SD values were also obtained in five measurements as the height of the ionization chamber survey meter was adjusted five times (80‐, 100‐, 120‐, 140‐, and 160‐cm heights).

### 2.5 Statistical analysis

The X‐ray parameters for digital cine acquisitions (tube potential, tube current, pulse width, spectral shaping filter, focal spot size, and the X‐ray beam field at FPIR) were automatically adjusted to obtain an image of diagnostic quality. Therefore, the SID and air kerma rate at the PERP in the right anterior oblique (RAO) and left anterior oblique (LAO) views were compared, with significant differences computed using the Wilcoxon rank‐sum test.^15^ Stray radiation is also affected by X‐ray parameters and the path of the X‐ray beam. Therefore, the stray radiations in the RAO and LAO views were compared, with significant differences computed using the Wilcoxon rank‐sum test.^15^

Furthermore, stray radiation reduces with increasing distance from the patient or the examination table regardless of the type of protection device used. To determine the strength of the relationship between stray radiation and height of the ionization chamber survey meter, Spearman's rank correlation coefficient was used.^16^ Moreover, because stray radiation is affected by the type of protection device used, the Friedman test was used to compare the degree to which stray radiation dose was reduced by the L‐shaped acrylic board, three types of DLAS, lead curtain, and combination.^17^ As a post hoc analysis, pairwise comparisons using Wilcoxon signed‐rank test with Holm corrections were used to compare the dose reductions among the L‐shaped acrylic board, three types of DLAS, lead curtain, and combination.^18^

Measurement variables (SID, air kerma rate at the PERP, stray radiation, and dose reduction) were reported as medians with interquartile range, and a *p* value of <0.05 was considered statistically significant. All statistical analyses were performed using the R software package for Windows version 3.5.2 (R Core Team (2018). R: A language and environment for statistical computing. R Foundation for Statistical Computing, Vienna, Austria).^19^

## 3 RESULTS

### 3.1 Measurements of the X‐ray parameters in the X‐ray tube angulation

Table 1 shows the X‐ray parameters used for digital acquisitions. Medians [first and third quartiles] of SID in all 10 X‐ray tube angulations (PA + RAO + LAO views), 4 RAO views, and 5 LAO views were 107.5 [105.0, 110.0], 107.5 [103.8, 110.0], and 110.0 [105.0, 110.0] cm, respectively. There was no significant difference in SID between the RAO and LAO views. Moreover, the median air kerma rates at the PERP in all X‐ray tube angulations, RAO views, and LAO views were 45.9 [28.3, 61.8], 30.4 [24.4, 42.3], and 61.8 [59.8, 138] mGy/min, respectively. Unlike the results for SID, the air kerma rate was significantly higher in LAO views than that in RAO views (*p* < 0.001).

### 3.2 Measurements of stray radiation and dose reduction

Tables 2a, 2b, 2c, 2d, 2e present the results of H*(10) measured at 80, 100, 120, 140, and 160 cm from the floor and the respective dose reductions using the protectors. When the protectors were not used, the H*(10) in all X‐ray tube angulations, RAO views, and LAO views was 7.24 [3.32, 18.8], 4.28 [3.13, 5.63], and 18.9 [12.3, 34.2] μSv/30 s, respectively. The stray radiation was significantly higher in LAO views than in RAO views (*p* < 0.001). Moreover, without the protectors, the median stray radiation measured in all X‐ray tube angulations was reduced with increasing height from the floor, and the correlation coefficient [95% confidence interval] was −0.128 [−0.214, −0.041] (*p* < 0.01).

**TABLE 2a:** Stray radiations measured at 80 cm from the floor and the dose reductions with protectors

	Stray radiations of digital acquisitions (H*(10): μSv/30 s) and the dose reductions (*R*_w/_: % in parenthesis)						
X‐ray tube angulation	No protections	L‐shaped acrylic board	DLAS with 0.5‐mm lead	DLAS with 0.75‐mm lead	DLAS with 1.0‐mm lead	Conventional lead curtain^a^	Combination^b^
PA 0°	3.30 (NA)	3.01 (8.7)	2.74 (17.0)	2.73 (17.3)	2.72 (17.6)	0.14 (95.8)	0.15 (95.5)
RAO 10°/CAU 30°	5.67 (NA)	5.22 (8.0)	4.94 (12.8)	4.93 (13.2)	4.91 (13.5)	0.26 (95.5)	0.27 (95.3)
RAO 30°/CAU 30°	9.11 (NA)	8.40 (7.8)	8.04 (11.8)	8.00 (12.2)	7.98 (12.4)	0.47 (94.8)	0.47 (94.8)
RAO 30°	4.54 (NA)	4.15 (8.7)	3.92 (13.7)	3.87 (14.8)	3.88 (14.5)	0.20 (95.7)	0.19 (95.9)
RAO 30°/CRA 30°	6.51 (NA)	5.90 (9.4)	5.59 (14.2)	5.58 (14.4)	5.61 (13.8)	0.26 (96.1)	0.25 (96.2)
LAO 10°/CRA 30°	10.3 (NA)	8.73 (15.6)	8.02 (22.5)	8.01 (22.6)	7.98 (22.9)	0.31 (97.0)	0.23 (97.8)
LAO 30°/CRA 30°	19.4 (NA)	15.8 (18.8)	14.3 (26.4)	14.1 (27.4)	14.2 (26.9)	0.40 (97.9)	0.34 (98.3)
LAO 45°/CRA 30°	51.6 (NA)	42.8 (17.2)	34.3 (33.6)	33.7 (34.7)	33.4 (35.3)	0.85 (98.4)	0.75 (98.5)
LAO 45°	20.5 (NA)	20.2 (1.4)	17.3 (15.4)	17.0 (16.8)	17.0 (16.8)	0.45 (97.8)	0.39 (98.1)
LAO 45°/CAU 30°	34.2 (NA)	30.6 (10.4)	26.6 (22.2)	26.4 (22.8)	26.4 (22.8)	6.59 (80.7)	4.63 (86.5)
Median stray radiation: H*(10)_w/o_ or H*(10)_w/_ [first and third quartiles]	9.73 [5.88, 20.2]	8.57 [5.39, 19.1]	8.03 [5.10, 16.6]	8.01 [5.09, 16.3]	7.98 [5.09, 16.3]	0.35 [0.26, 0.47]	0.30 [0.23, 0.45]
Median dose reduction in all views: *R*_w/_ [first and third quartiles]	NA	9.1 [8.2, 14.3]	16.2 [13.8, 22.4]	17.1 [14.5, 22.8]	17.2 [14.0, 22.9]	96.0 [95.6, 97.6]	96.1 [95.4, 98.0]
Median dose reduction in RAO views: *R*_w/_ [first and third quartiles]	NA	8.4 [8.0, 8.9]	13.3 [12.6, 13.8]	13.8 [13.0, 14.5]	13.7 [13.2, 14.0]	95.6 [95.3, 95.8]	95.6 [95.2, 96.0]
Median dose reduction in LAO views: *R*_w/_ [first and third quartiles]	NA	15.6 [10.4, 17.2]	22.5 [22.2, 26.4]	22.8 [22.6, 27.4]	22.9 [22.8, 26.9]	97.8 [97.0, 97.9]	98.1 [97.8, 98.3]

Abbreviations: CAU, caudal; CRA, cranial; DLAS, detachable lead arm support; LAO, left anterior oblique; NA, not applicable; PA, posterior–anterior; RAO, right anterior oblique.

^a^ The conventional 0.5‐mm lead curtain suspended from the table side rail.

^b^ The combination consists of DLAS with 0.5‐mm lead and lead curtain.

**TABLE 2b:** Stray radiations measured at 100 cm from the floor and the dose reductions with protectors

	Stray radiations of digital acquisitions (H*(10): μSv/30 s) and the dose reductions (*R*_w/_: % in parenthesis)						
X‐ray tube angulation	No protections	L‐shaped acrylic board	DLAS with 0.5‐mm lead	DLAS with 0.75‐mm lead	DLAS with 1.0‐mm lead	Conventional lead curtain^a^	Combination^b^
PA 0°	3.14 (NA)	2.89 (8.2)	1.82 (42.0)	1.83 (41.7)	1.84 (41.4)	0.14 (95.6)	0.12 (96.2)
RAO 10°/CAU 30°	4.99 (NA)	4.57 (8.5)	2.95 (41.0)	2.95 (41.0)	2.95 (41.0)	0.25 (95.1)	0.25 (95.1)
RAO 30°/CAU 30°	8.52 (NA)	7.75 (9.0)	5.50 (35.5)	5.52 (35.3)	5.50 (35.5)	0.44 (94.8)	0.44 (94.8)
RAO 30°	4.02 (NA)	3.67 (8.6)	2.84 (29.4)	2.85 (29.2)	2.88 (28.4)	0.19 (95.3)	0.19 (95.3)
RAO 30°/CRA 30°	5.62 (NA)	5.14 (8.4)	4.36 (22.3)	4.42 (21.2)	4.39 (21.8)	0.29 (94.9)	0.28 (95.1)
LAO 10°/CRA 30°	8.33 (NA)	7.08 (15.0)	4.32 (48.1)	4.44 (46.7)	4.35 (47.8)	0.32 (96.2)	0.22 (97.4)
LAO 30°/CRA 30°	15.9 (NA)	12.3 (22.4)	5.46 (65.6)	5.55 (65.0)	5.50 (65.3)	0.43 (97.3)	0.35 (97.8)
LAO 45°/CRA 30°	70.6 (NA)	54.2 (23.3)	19.5 (72.4)	19.4 (72.5)	18.9 (73.2)	1.07 (98.5)	0.78 (98.9)
LAO 45°	18.9 (NA)	17.5 (7.3)	5.98 (68.4)	5.93 (68.6)	5.91 (68.8)	0.48 (97.4)	0.35 (98.2)
LAO 45°/CAU 30°	33.8 (NA)	29.3 (13.4)	12.9 (61.8)	12.7 (62.4)	12.8 (62.1)	6.59 (80.5)	2.04 (94.0)
Median stray radiation: H*(10)_w/o_ or H*(10)_w/_ [first and third quartiles]	8.43 [5.15, 18.1]	7.42 [4.71, 16.2]	4.91 [3.29, 5.86]	4.98 [3.31, 5.83]	4.94 [3.30, 5.81]	0.37 [0.26, 0.47]	0.31 [0.22, 0.42]
Median dose reduction in all views: *R*_w/_ [first and third quartiles]	NA	8.8 [8.4, 14.6]	45.1 [36.9, 64.7]	44.2 [36.7, 64.4]	44.6 [36.9, 64.5]	95.5 [95.0, 97.0]	95.8 [95.1, 97.7]
Median dose reduction in RAO views: *R*_w/_ [first and third quartiles]	NA	8.6 [8.5, 8.7]	32.5 [27.6, 36.9]	32.3 [27.2, 36.7]	32.0 [26.8, 36.9]	95.0 [94.9, 95.2]	95.1 [95.0, 95.2]
Median dose reduction in LAO views: *R*_w/_ [first and third quartiles]	NA	15.0 [13.4, 22.4]	65.6 [61.8, 68.4]	65.0 [62.4, 68.6]	65.3 [62.1, 68.8]	97.3 [96.2, 97.4]	97.8 [97.4, 98.2]

Abbreviations: CAU, caudal; CRA, cranial; DLAS, detachable lead arm support; LAO, left anterior oblique; NA, not applicable; PA, posterior–anterior; RAO, right anterior oblique.

^a^ The conventional 0.5‐mm lead curtain suspended from the table side rail.

^b^ The combination consists of DLAS with 0.5‐mm lead and lead curtain.

**TABLE 2c:** Stray radiations measured at 120 cm from the floor and the dose reductions with protectors

	Stray radiations of digital acquisitions (H*(10): μSv/30 s) and the dose reductions (*R*_w/_: % in parenthesis)						
X‐ray tube angulation	No protections	L‐shaped acrylic board	DLAS with 0.5‐mm lead	DLAS with 0.75‐mm lead	DLAS with 1.0‐mm lead	Conventional lead curtain^a^	Combination^b^
PA 0°	3.24 (NA)	3.02 (6.8)	1.74 (46.3)	1.75 (46.0)	1.74 (46.3)	0.32 (90.1)	0.20 (93.8)
RAO 10°/CAU 30°	5.16 (NA)	4.38 (15.1)	1.88 (63.6)	1.58 (69.4)	1.52 (70.5)	0.54 (89.5)	0.33 (93.6)
RAO 30°/CAU 30°	6.20 (NA)	4.87 (21.5)	1.24 (80.0)	1.24 (80.0)	1.19 (80.8)	1.04 (83.2)	0.59 (90.5)
RAO 30°	3.22 (NA)	2.84 (11.8)	1.78 (44.7)	1.84 (42.9)	1.83 (43.2)	0.51 (84.2)	0.29 (91.0)
RAO 30°/CRA 30°	3.36 (NA)	3.11 (7.4)	2.33 (30.7)	2.34 (30.4)	2.36 (29.8)	0.61 (81.8)	0.39 (88.4)
LAO 10°/CRA 30°	7.97 (NA)	6.94 (12.9)	4.26 (46.5)	4.46 (44.0)	4.43 (44.4)	0.74 (90.7)	0.37 (95.4)
LAO 30°/CRA 30°	14.5 (NA)	12.0 (17.2)	4.18 (71.2)	4.56 (68.6)	4.47 (69.2)	1.72 (88.1)	0.60 (95.9)
LAO 45°/CRA 30°	65.2 (NA)	52.9 (18.9)	10.2 (84.4)	10.2 (84.4)	10.2 (84.4)	40.5 (37.9)	4.05 (93.8)
LAO 45°	18.5 (NA)	18.4 (0.5)	4.0 (78.4)	3.99 (78.4)	3.96 (78.6)	11.6 (37.3)	1.03 (94.4)
LAO 45°/CAU 30°	36.4 (NA)	32.7 (10.2)	11.5 (68.4)	11.1 (69.5)	11.3 (69.0)	8.51 (76.6)	1.83 (95.0)
Median stray radiation: H*(10)_w/o_ or H*(10)_w/_ [first and third quartiles]	7.09 [3.81, 17.5]	5.91 [3.43, 16.8]	3.17 [1.75, 4.24]	3.17 [1.77, 4.54]	3.16 [1.76, 4.46]	0.89 [0.56, 6.81]	0.49 [0.34, 0.92]
Median dose reduction in all views: *R*_w/_ [first and third quartiles]	NA	12.4 [8.1, 16.7]	66.0 [46.4, 76.6]	69.0 [44.5, 76.2]	69.1 [44.9, 76.6]	83.7 [77.9, 89.2]	93.8 [91.7, 94.9]
Median dose reduction in RAO views: *R*_w/_ [first and third quartiles]	NA	13.5 [10.7, 16.7]	54.2 [41.2, 67.7]	56.2 [40.0, 72.1]	56.9 [40.0, 73.1]	83.7 [82.9, 85.5]	90.8 [90.0, 91.7]
Median dose reduction in LAO views: *R*_w/_ [first and third quartiles]	NA	12.9 [10.2, 17.2]	71.2 [68.4, 78.4]	69.5 [68.6, 78.4]	69.2 [69.0, 78.6]	76.6 [37.9, 88.1]	95.0 [94.4, 95.4]

Abbreviations: CAU, caudal; CRA, cranial; DLAS, detachable lead arm support; LAO, left anterior oblique; NA, not applicable; PA, posterior–anterior; RAO, right anterior oblique.

^a^ The conventional 0.5‐mm lead curtain suspended from the table side rail.

^b^ The combination consists of DLAS with 0.5‐mm lead and lead curtain.

**TABLE 2d:** Stray radiations measured at 140 cm from the floor and the dose reductions with protectors

	Stray radiations of digital acquisitions (H*(10): μSv/30 s) and the dose reductions (*R*_w/_: % in parenthesis)						
X‐ray tube angulation	No protections	L‐shaped acrylic board	DLAS with 0.5‐mm lead	DLAS with 0.75‐mm lead	DLAS with 1.0‐mm lead	Conventional lead curtain^a^	Combination^b^
PA 0°	2.59 (NA)	2.24 (13.5)	0.93 (64.1)	0.89 (65.6)	0.85 (67.2)	1.03 (60.2)	0.61 (76.4)
RAO 10°/CAU 30°	3.24 (NA)	2.62 (19.1)	1.14 (64.8)	1.13 (65.1)	1.14 (64.8)	2.01 (38.0)	1.00 (69.1)
RAO 30°/CAU 30°	5.03 (NA)	4.21 (16.3)	2.08 (58.6)	2.08 (58.6)	2.07 (58.8)	4.11 (18.3)	1.84 (63.4)
RAO 30°	2.32 (NA)	2.07 (10.8)	1.73 (25.4)	1.77 (23.7)	1.75 (24.6)	1.69 (27.2)	1.44 (37.9)
RAO 30°/CRA 30°	2.84 (NA)	2.73 (3.9)	2.36 (16.9)	2.36 (16.9)	2.36 (16.9)	2.00 (29.6)	1.88 (33.8)
LAO 10°/CRA 30°	6.18 (NA)	5.50 (11.0)	3.85 (37.7)	3.88 (37.2)	3.89 (37.1)	2.87 (53.6)	1.67 (73.0)
LAO 30°/CRA 30°	12.3 (NA)	10.5 (14.6)	4.38 (64.4)	4.55 (63.0)	4.51 (63.3)	9.24 (24.9)	2.57 (79.1)
LAO 45°/CRA 30°	49.8 (NA)	42.9 (13.9)	9.38 (81.2)	9.62 (80.7)	9.11 (81.7)	39.1 (21.5)	9.10 (81.7)
LAO 45°	15.9 (NA)	16.7 (−5.0)	5.33 (66.5)	5.33 (66.5)	5.29 (66.7)	15.5 (2.5)	5.14 (67.7)
LAO 45°/CAU 30°	33.6 (NA)	30.9 (8.0)	11.5 (65.8)	11.4 (66.1)	11.4 (66.1)	26.8 (20.2)	5.27 (84.3)
Median stray radiation: H*(10)_w/o_ or H*(10)_w/_ [first and third quartiles]	5.61 [2.94, 15.0]	4.86 [2.65,15.2]	3.11 [1.82, 5.09]	3.12 [1.85, 5.14]	3.13 [1.83, 5.10]	3.49 [2.00, 13.9]	1.86 [1.50, 4.50]
Median dose reduction in all views: *R*_w/_ [first and third quartiles]	NA	12.3 [8.7, 14.4]	64.3 [42.9, 65.6]	64.1 [42.6, 66.0]	64.1 [42.5, 66.6]	26.1 [20.5, 35.9]	71.1 [64.5, 78.4]
Median dose reduction in RAO views: *R*_w/_ [first and third quartiles]	NA	13.6 [9.1, 17.0]	42.0 [23.3, 60.2]	41.2 [22.0, 60.2]	41.7 [22.7, 60.3]	28.4 [25.0, 31.7]	50.7 [36.9, 64.8]
Median dose reduction in LAO views: *R*_w/_ [first and third quartiles]	NA	11.0 [8.0, 13.9]	65.8 [64.4, 66.5]	66.1 [63.0, 66.5]	66.1 [63.3, 66.7]	21.5 [20.2, 24.9]	79.1 [73.0, 81.7]

Abbreviations: CAU, caudal; CRA, cranial; DLAS, detachable lead arm support; LAO, left anterior oblique; NA, not applicable; PA, posterior–anterior; RAO, right anterior oblique.

^a^ The conventional 0.5‐mm lead curtain suspended from the table side rail.

^b^ The combination consists of DLAS with 0.5‐mm lead and lead curtain.

**TABLE 2e:** Stray radiations measured at 160 cm from the floor and the dose reductions with protectors

	Stray radiations of digital acquisitions (H*(10): μSv/30 s) and the dose reductions (*R*_w/_: % in parenthesis)						
X‐ray tube angulation	No protections	L‐shaped acrylic board	DLAS with 0.5‐mm lead	DLAS with 0.75‐mm lead	DLAS with 1.0‐mm lead	Conventional lead curtain^a^	Combination^b^
PA 0°	1.98 (NA)	1.72 (13.5)	1.00 (49.5)	1.00 (49.5)	1.00 (49.5)	1.49 (25.0)	0.96 (51.6)
RAO 10°/CAU 30°	3.30 (NA)	2.73 (17.2)	1.66 (49.5)	1.65 (49.8)	1.63 (50.5)	2.69 (18.5)	1.58 (52.0)
RAO 30°/CAU 30°	2.43 (NA)	2.39 (1.7)	2.03 (16.6)	2.05 (15.7)	2.05 (15.7)	1.95 (19.6)	1.91 (21.3)
RAO 30°	1.69 (NA)	1.65 (1.8)	1.51 (10.4)	1.50 (11.0)	1.50 (11.0)	1.35 (19.6)	1.30 (22.7)
RAO 30°/CRA 30°	2.15 (NA)	2.05 (4.8)	1.86 (13.5)	1.86 (13.5)	1.86 (13.5)	1.62 (24.5)	1.54 (28.4)
LAO 10°/CRA 30°	4.75 (NA)	4.37 (8.0)	3.76 (20.9)	3.80 (20.0)	3.78 (20.4)	3.56 (25.2)	2.54 (46.5)
LAO 30°/CRA 30°	9.64 (NA)	8.75 (9.2)	5.21 (46.0)	5.35 (44.5)	5.38 (44.3)	8.65 (10.3)	5.04 (47.7)
LAO 45°/CRA 30°	39.9 (NA)	35.9 (10.1)	15.2 (61.9)	15.3 (61.7)	15.5 (61.1)	31.2 (21.8)	15.1 (62.2)
LAO 45°	13.7 (NA)	15.4 (−12.0)	8.24 (40.1)	7.63 (44.5)	7.68 (44.1)	13.5 (1.5)	8.17 (40.6)
LAO 45°/CAU 30°	29.7 (NA)	28.9 (2.4)	15.4 (48.1)	15.7 (47.0)	15.6 (47.4)	26.6 (10.5)	15.3 (48.4)
Median stray radiation: H*(10)_w/o_ or H*(10)_w/_ [first and third quartiles]	4.03 [2.22, 12.7]	3.55 [2.13, 13.7]	2.89 [1.71, 7.48]	2.93 [1.71, 7.06]	2.91 [1.69, 7.10]	3.12 [1.71, 12.3]	2.23 [1.55, 7.39]
Median dose reduction in all views: *R*_w/_ [first and third quartiles]	NA	6.4 [2.0, 9.9]	43.1 [17.7, 49.2]	44.5 [16.8, 48.9]	44.2 [16.9, 49.0]	19.6 [12.5, 23.8]	47.1 [31.5, 50.8]
Median dose reduction in RAO views: *R*_w/_ [first and third quartiles]	NA	3.3 [1.8, 7.9]	15.1 [12.7, 24.8]	14.6 [12.9, 24.2]	14.6 [12.9, 24.4]	19.6 [19.3, 20.8]	25.6 [22.4, 34.3]
Median dose reduction in LAO views: *R*_w/_ [first and third quartiles]	NA	8.0 [2.4, 9.2]	46.0 [40.1, 48.1]	44.5 [44.5, 47.0]	44.3 [44.1, 47.4]	10.5 [10.3, 21.8]	47.7 [46.5, 48.4]

Abbreviations: CAU, caudal; CRA, cranial; DLAS, detachable lead arm support; LAO, left anterior oblique; NA, not applicable; PA, posterior–anterior; RAO, right anterior oblique.

^a^ The conventional 0.5‐mm lead curtain suspended from the table side rail.

^b^ The combination consists of DLAS with 0.5‐mm lead and lead curtain.

Median dose reductions of stray radiation using the L‐shaped acrylic board were 9.0%, 8.8%, 12.4%, 12.3%, and 6.4% at 80‐, 100‐, 120‐, 140‐, and 160‐cm heights, respectively. Dose reduction using DLAS with a 0.5‐mm lead was almost identical to that using DLAS with 0.75‐ and 1.0‐mm leads; mean dose reductions using these three DLASs increased to 16.2%, 45.1%, 66.0%, 64.2%, and 43.0% at 80‐, 100‐, 120‐, 140‐, and 160‐cm heights, respectively. Although each DLAS facilitated a greater dose reduction than the L‐shaped acrylic board at all heights (*p* < 0.05), there were no significant differences among the dose reductions using these three DLASs between the heights of 100 and 160 cm. The dose reduction using the DLAS with 0.5‐mm lead was lower than that using the DLAS with 0.75‐ and 1.0‐mm lead at 80 cm (*p* < 0.05, the difference of the median was 0.9%). Similarly, dose reductions using the conventional lead curtain were 95.9%, 95.5%, 83.7%, 26.0%, and 19.6% at 80‐, 100‐, 120‐, 140‐, and 160‐cm heights, respectively. The conventional lead curtain resulted in a greater dose reduction than each DLAS used at 80‐ and 100‐cm heights (*p* < 0.05), whereas no statistically significant differences were observed at ≥120‐cm heights. Furthermore, the combination of DLAS with 0.5‐mm lead and lead curtain could increase dose reductions to 96.0%, 95.8%, 93.8%, 71.1%, and 47.1%, respectively. There was a greater dose reduction using the combination than using the lead curtain only at 120‐, 140‐, and 160‐cm heights (*p* < 0.05), whereas no statistically significant differences were observed at 80‐ and 100‐cm heights. Furthermore, the median dose reductions using all DLAS were higher in LAO views than in RAO views (*p* < 0.01). However, there were no significant differences in dose reductions between the RAO and LAO views using the L‐shaped acrylic board, lead curtain, and combination.

## 4 DISCUSSION

In this study, stray radiation was measured to assess the protection efficiency of the L‐shaped acrylic board, DLAS with 0.5‐, 0.75‐, and 1.0‐mm leads, lead curtain, and the combination of DLAS with 0.5‐mm lead and lead curtain. Although the DLAS would not be used alone in the clinical setting, dose reduction using each of these protectors must be measured to clarify their protection efficiencies. Our findings demonstrate significantly higher dose reductions for all DLAS than for the L‐shaped acrylic board, whereas no remarkable differences were observed among the three types of DLAS. Moreover, the dose reduction using the lead curtain was limited to heights ≤100 cm. In contrast, DLAS was effective for reducing stray radiation at heights ≥120 cm. Therefore, the combination of the DLAS and lead curtain would be an appealing radioprotective tool in PCI.

Typically, methods for reducing occupational radiation dose in a catheterization laboratory include wearing protective gears, optimizing the use of the cardiovascular angiographic system, and shielding stray radiation. Many types of radioprotective gears, such as protective apron, thyroid shield, leaded face shield, and leg protection, can be used to reduce the occupational radiation dose^8^; however, their heavyweights have been linked to orthopedic complications in cardiologists.^20^ Ideally, a suspended radioprotective system should be used to address this issue^21^; however, such systems are not used widely in the cardiology community. The optimized use of the cardiovascular angiographic system is also imperative for reducing occupational radiation dose. Reducing fluoroscopy and digital acquisition time, optimal table positioning, collimating the radiation field to the region of interest, a low fluoroscopy/digital acquisition frame rate, and using a thick copper filtration would collectively reduce the occupational radiation dose.^8^, ^22^, ^23^ Moreover, because biplane imaging for PCI is related to an increased occupational radiation dose, a monoplane imaging should be considered for advanced radiation protection in catheterization laboratories.^24^ Furthermore, shielding the stray radiation right next to the patient is an important technique to reduce the occupational radiation dose. The lead curtain, ceiling‐suspended lead shield, and lead‐free drape have been used in the cardiology community to reduce the stray radiation.^9^ The DLAS, in conjunction with these protective devices, can effectively reduce stray radiation without interfering with the clinical environments.

The SID in LAO views with steep angle might be larger than in RAO views to avoid collisions with the patient. However, as shown in Table 1, there was no statistically significant difference in the SID groups between the RAO and LAO views. In contrast, the air kerma rates measured at the PERP and the stray radiation were higher in the LAO views than in the RAO views (*p* < 0.001). This is because the primary X‐rays in the LAO views are more penetrating in the long path of the patient's heart than those in the RAO views.^10^

As anticipated, the dose reductions using the L‐shaped acrylic board were lower than those using all DLAS at all heights. Interestingly, the L‐shaped acrylic board increased the stray radiation doses measured at 140‐ and 160‐cm heights at a 45° angle of the LAO view. This is attributable to the scattered X‐rays from the L‐shaped acrylic board, indicating that the L‐shaped acrylic board should be covered with the detachable water‐resistant cover encasing 0.5‐, 0.75‐, or 1.0‐mm lead.

The dose reduction using the DLAS with 0.5‐mm lead was almost identical to those using DLAS with 0.75‐ and 1.0‐mm leads, as the stray radiation at 50 keV could be reduced by 99.0% when using the DLAS with 0.5‐mm lead.^25^ Furthermore, because the maximum tube potential was 75 kVp in this study, the averaged energy of the stray radiation would be lower than 50 keV, which is almost entirely attenuated if the scattered X‐rays interact with the DLAS.^26^

The dose reductions using the lead curtain were higher than those using DLAS at 80‐ and 100‐cm heights (*p* < 0.05), whereas no statistically significant differences were observed at ≥120‐cm heights. This result was anticipated because when the patient lies on the examination table at the PERP, the lead curtain is suspended from the table side rail at a height of 97.5 cm from the floor. In addition, the combination of DLAS with 0.5‐mm lead and lead curtain demonstrated a greater dose reduction than the lead curtain at 120‐, 140‐, and 160‐cm (*p* < 0.05) heights from the ground, whereas no statistically significant differences were observed at 80‐ and 100‐cm heights. This finding indicates that the combination of the DLAS and lead curtain would be an appealing radioprotective tool in PCI.

The median dose reductions using all DLAS were higher in the LAO views than in the RAO views (*p* < 0.01). The incident X‐ray beam area in the LAO view is distributed on the skin surface of the right side of the patient's back.^27^ Therefore, a substantial amount of scattered X‐rays originates from the DLAS surroundings. As described in Section 1, it is imperative to place the radioprotective device right next to the patient as effectively as possible to reduce stray radiation. Because stray radiation is greater in the LAO view than in the RAO view, DLAS could become a promising radioprotective device.

This study had some limitations. First, Schueler et al. reported the sophisticated technique of measuring stray radiation with six 180‐cm^3^ ionization chambers in a row.^28^ Based on their concept, the air kerma should ideally be measured at the heights between 80 and 160 cm in 10‐cm increments to determine the change in stray radiation dose distribution. Theoretically, a small chamber would be preferable to better characterize the stray radiation resulting from the protectors in steep dose gradient areas. We have access to a 6‐cm^3^ ionization chamber. Unfortunately, the response was too low to measure the stray radiation in PCI. Second, air kerma should be used to calculate the stray radiation dose reduction. As described in Section 2, the ionization chamber survey meter has a relatively large energy dependence (15%) to emulate the conversion coefficients from air kerma to H*(10) (the operational quantity for assessing effective dose). To measure the air kerma, the ionization chamber is frequently used because its energy dependence is lower than that of the ionization chamber survey meter. The dose reduction using the protectors was calculated as H*(10) measurements in this study. Thus, small differences would have been considered among the dose reductions calculated by the H*(10) and the air kerma. Third, the ceiling‐suspended lead shield was not used in this study because the reduction of stray radiation was expected to depend on the shield location, and it is difficult to replicate the identical positions. Moreover, the cardiovascular angiographic system was not equipped with the radioprotective drape.^9^ Combining the DLAS, lead curtain, ceiling‐suspended lead shield, and radioprotective drape can further reduce the stray radiation. Fourth, the DLAS was placed right next to the female anthropomorphic phantom, with a 13‐cm separation (typical arm thickness and space for movement) to emulate the right arm space. The stray radiation dose reductions using the DLAS might be reduced with increasing arm thickness, particularly at a 160‐cm height of the ionization chamber survey meter. However, because scattered X‐rays can be absorbed in thick arms, stray radiation dose reductions using the DLAS might be unaffected. Thus, further studies are warranted to determine whether the thickness of the patient's arm can lead to changes in the radioprotective efficiency of DLAS.

## 5 CONCLUSION

We developed a novel DLAS to reduce stray radiation without interfering with the clinical environment in the catheterization laboratory. The DLAS consists of an L‐shaped acrylic board and a detachable water‐resistant cover encasing the 0.5‐, 0.75‐, and 1.0‐mm leads. The dose reductions using all DLAS were significantly higher than the dose reduction using the L‐shaped acrylic board, and no remarkable differences were observed among the three types of DLAS examined. Moreover, the dose reductions using DLAS were effective in the LAO views where the stray radiation was relatively high. In addition, the dose reduction using the lead curtain was limited to heights ≤100 cm; thus, the combination of the DLAS and lead curtain would be an appealing method for reducing stray radiation.

## AUTHOR CONTRIBUTION

Atsushi Fukuda, Ph.D, Conception and design of the study, analysis and interpretation of data, collection and assembly of data, drafting of the article, and final approval of the article. Nao Ichikawa, MSc, Conception and design of the study, analysis and interpretation of data, collection and assembly of data, and final approval of the article. Takuma Hayashi, RT, Conception and design of the study, analysis and interpretation of data, collection and assembly of data, and final approval of the article. Pei‐Jan P. Lin, Ph.D, Conception and design of the study, analysis and interpretation of data, critical revising, final approval of the article. Kosuke Matsubara, Ph.D, Conception and design of the study, analysis and interpretation of data, and final approval of the article.

## CONFLICT OF INTEREST

Atsushi Fukuda receives a research grant from Hoshina Corporation for research in the detachable lead arm support (DLAS).
